# Study on the Cutting Performance of Micro Textured Tools on Cutting Ti-6Al-4V Titanium Alloy

**DOI:** 10.3390/mi11020137

**Published:** 2020-01-25

**Authors:** Kairui Zheng, Fazhan Yang, Na Zhang, Qingyu Liu, Fulin Jiang

**Affiliations:** School of Mechanical and Automotive Engineering, National and local joint Engineering Research Center for efficient resource utilization of metallurgical slag, Qingdao University of Technology, Qingdao 266520, China; kairuiz@foxmail.com (K.Z.); qutzhangn@foxmail.com (N.Z.); qingyu-liu@foxmail.com (Q.L.); sdujiangfulin@gmail.com (F.J.)

**Keywords:** textured tool, titanium alloy, cutting performance, cutting force, cutting fluid

## Abstract

Titanium alloys are widely used in various fields, but their machinability is poor because the chip would easily adhere to the tool surface during cutting, causing poor surface quality and tool wear. To improve the cutting performance of titanium alloy Ti-6Al-4V, experiments were conducted to investigate the effect of micro textured tool on the cutting performances. The cemented carbide tools whose rake faces were machined with line, rhombic, and sinusoidal groove textures with 10% area occupancy rates were adopted as the cutting tools. The effects of cutting depth and cutting speed on feed force and main cutting force were discussed based on experimental results. The results show that the cutting force produced by textured tools is less than that produced by non-textured tools. Under different cutting parameters, the best cutting performance can be obtained by using sinusoidal textured tools among the four types of tools. The wear of micro textured tools is significantly lower than that of non-textured tools, due to a continuous lubrication film between the chip and the rake face of the tool that can be produced because the micro texture can store and replenish lubricant. The surface roughness obtained using the textured tool is better than that using the non-textured tool. The surface roughness Ra can be reduced by 35.89% when using sinusoidal textured tools. This study is helpful for further improving the cutting performance of cemented carbide tools on titanium alloy and prolonging tool life.

## 1. Introduction

In recent years, titanium alloy has been widely used in aeronautics and astronautics, and medical, chemical, automotive, and other fields because of its excellent comprehensive properties [1,2,3,4]. However, titanium alloy is one of the difficult-to-cut materials due to its high hardness, high strength, low elastic modulus, and high chemical activity at high temperature [5,6,7]. During the cutting process, there are a series of problems such as high cutting heat and sharp friction between the chip and the tool, causing high temperature and pressure in the cutting zone [8,9,10]. As a result, the chip easily adheres to the tool surface, causing tool wear and reducing tool life.

Lots of research has been carried out to improve tool life and cutting performance. It was found that tools covered with a durable ceramic coating can reduce tool wear and improve tool cutting performance [11]. Cryogenic treatment of the cutting tool was proven to be an effective method to improve the cutting performance [12]. The application of cutting fluid flushing during the cutting process has also been demonstrated to have a beneficial influence on tool life and the cutting performance [13]. However, the cutting performance still cannot meet the requirements of mass production, and the cost is still high. In the field of tribology, a concept of micro texture is put forward. The definition of micro texture technology is to use certain processing methods to manufacture craters, grooves, or protrusions on the friction pair surface to improve tribological properties [14]. At present, laser processing technology, electron beam processing technology, LIGA (Lithographie, Galvanoformung, Abformung) technology, micro EDM (electrical discharge machining) technology, and micro turning technology are widely used to process micro textures on the cutting tool [15]. Laser processing technology has become the preferred method for processing micro textures on the cutting tool, owing to its advantage of high energy density, high machining accuracy, and high machining efficiency [16].

Many studies have reported that compared with non-textured tools, the comprehensive performance of micro textured tools is better. Bai et al. [17] proposed a new method to quantitatively simulate the micro structure evolution of subsurface damage of titanium alloy components under micro-scale cutting. They found that the existence of micro textures on the tool surface could significantly reduce tool wear and improve cutting performance. Singh et al. [18] indicated that the existence of micro textures on high speed steel tools could improve the cutting performance effectively. Pang et al. [19] demonstrated that the micro texture can store cutting fluid during the cutting process, therefore, the friction properties of the tool surface could be improved, and the adhesive wear could be reduced. Olleak et al. [20] found that better stress distribution and lower wear depth can be obtained when using textured tools. Feng et al. [21] indicated that the cutting force and cutting temperature could be significantly reduced when a textured tool was used. As a conclusion, the textures on the tool surface can effectively reduce the tool–chip contact surface, reduce cutting temperature and cutting force, store lubricant, improve the contact state, and decrease the adhesion phenomenon [22,23,24,25]. However, the effect of sinusoidal textures and rhombic micro textures of tools on the cutting performance of titanium alloy is still unclear, which is necessary to be studied to further improve the cutting efficiency and surface quality of titanium alloy.

In this paper, experiments were conducted to investigate the effect of micro textured tools on cutting performance. The carbide tools whose rake faces were machined with line, rhombic, and sinusoidal groove textures with 10% area occupancy rates were adopted as the cutting tools. The effects of cutting depth and cutting speed on feed force, main cutting force, and surface roughness were discussed based on experimental results. 

## 2. Materials and Methods 

### 2.1. The Preparation of Samples

A titanium alloy bar (Ti-6Al-4V) with a size of Φ100 mm × 150 mm was adopted as the workpiece in the experiment. Cemented carbide has been widely used as tool material because of its excellent cutting performance, and a YG8 tool is adopted in this study. Its physical characteristics are shown in Table 1. The geometric parameters of the tool are shown in Table 2. 

The cemented carbide YG8 tool was pretreated to reduce the surface roughness, before the surface texture was machined. Firstly, the cemented carbide tool was ground roughly, and then metallographic sandpapers were used to perform semi-fine grinding and fine grinding to make the surface roughness Ra be less than 0.1 μm. Finally, the YG8 tool was ultrasonically cleaned for 10 min, and dried in a self-controlled infrared oven. 

**Figure 1:**
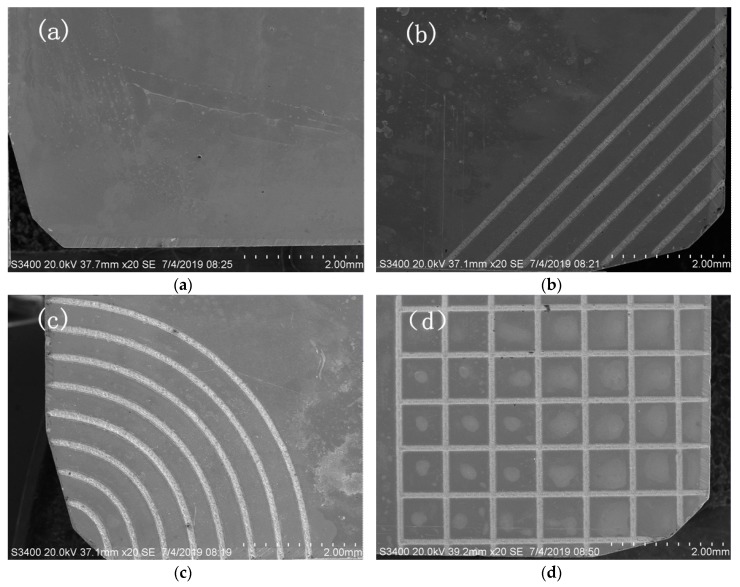
Surface micrographs of the four types of tools. (**a**) Non-textured tool; (**b**) Line textured tool; (**c**) Sinusoidal textured tool; (**d**) Rhombic textured tool.

Interpublic Group of Companies laser processing technology (YLPN-1-100-200-R, IPG, New York, NY, USA) was used to machine the micro textures on the rake face of the YG8 tool. Processing parameters were determined based on preliminary trials, which are shown in Table 3. Three types of groove textures (line groove, sinusoidal groove, and rhombic groove) with the width of 159.599 μm and the depth of 14.59 μm were fabricated. The surface micrographs of the four tools used in this study are shown in Figure 1.

### 2.2. Experimental Equipment and Methods

Cutting experiments were conducted on a CA6140 lathe (Zaozhuang puche Machine Tool Co., Ltd, Zaozhuang, China). During the cutting process, the titanium alloy bar rotated with the main shaft to perform the main movement. The vertical and horizontal movement of the tool was the feed movement. The cutting forces were measured by a YDC-Ⅲ 89A (designed by Dalian University of Technology) three-direction piezoelectric turning dynamometer. The experiments were tested by the single factor method, and the cutting parameters are shown in Table 4. The cutting fluid used in the experiment was ZJ-846 (Guangzhou ZhiJing Technology Co., Ltd., Guangzhou, China) concentrated cutting fluid (produced by Guangzhou ZhiJing Technology Co., Ltd., Guangzhou, China), which was diluted in the proportion of 1:20. Each experiment was conducted at least three times to ensure the repeatability. 

## 3. Results and Discussion

### 3.1. Effect of Cutting Parameters on Cutting Forces

**Figure 2:**
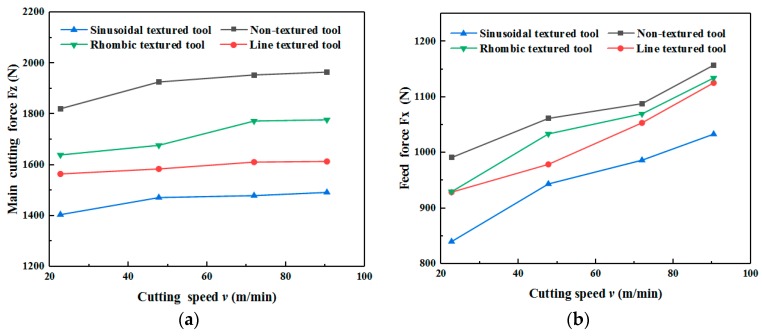
Main cutting force and feed force of tools at different cutting speeds (*a_p_* = 0.3 mm). (**a**) Main cutting force; (**b**) Feed force.

Figure 2 shows the feed force and main cutting force of four types of tools at different cutting speeds. It can be found from Figure 2 that the main cutting force and feed force increase with the cutting speed, regardless of tool type. This is because the built-up edge generated on the rake face decreased as the cutting speed increased, which increased the cutting deformation of the titanium alloy, resulting in the increase of cutting force. Therefore, the cutting force shows an increasing trend as the cutting speed increases.

It can also be found from Figure 2 that the cutting force of textured tools is less compared to non-textured tools. Among the four types of tools, sinusoidal textured tools can produce the least cutting force. The cutting force can be expressed as [26,27]:

(1) $$F_{z}=a_{w}l_{f}\tau_{c}(\sin\gamma_{o}+\frac{\cos\gamma_{o}}{\tan\beta })$$

(2) $$F_{x}=a_{w}l_{f}\tau_{c}(\cos\gamma_{o}-\frac{\sin\gamma_{o}}{\tan\beta })\cos(\varphi_{r}+\varphi_{\lambda })$$

where, *F_z_* represents the main cutting force, *F_x_* represents the feed force, *l_f_* represents the contact length of the tool and chip, *a*_w_ represents the cutting width, $\tau_{c}$ represents the average shear force of the tool–chip contact surface, $\gamma_{0}$ represents the rake angle of the tool, *β* represents the friction angle, *φ_r_* represents the residual deviation angle, and $\varphi_{\lambda }$ represents the chip outflow angle.

It can be found from Equations (1) and (2) that the main cutting force is proportional to cutting width, the contact length of the tool and chip, and the average shear force. The groove textures on the rake face of the tool act as chip breakers, therefore, the micro texture causes a smaller tool–chip contact area and shorter contact length. Under the effect of cutting force, friction force, and cutting heat, the cutting fluid would be coated on the contact surface between the tool and chip, forming a lubricant film. The average shear force and cutting friction force are reduced owing to the lubricant film. As a result, the main cutting force and feed force decrease correspondingly. Compared with non-textured tools, the main cutting force of line, sinusoidal, and rhombic textured tools can be reduced by 17.88%, 24.06%, and 12.93%. The feed force can be reduced by 7.79%, 15.25%, and 6.20%, respectively. 

**Figure 3:**
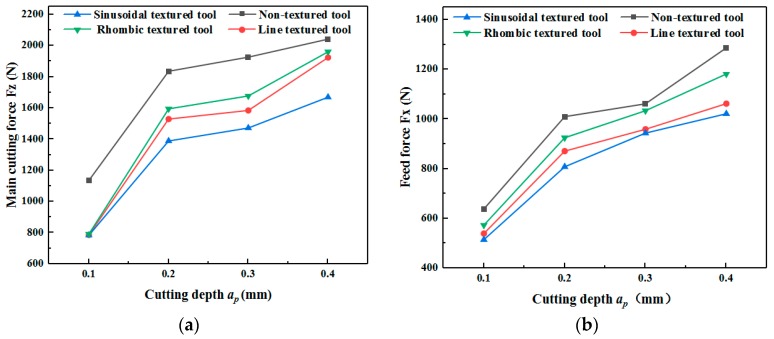
Main cutting force and feed force of tools at different cutting depths (*v* = 47.7 m/min). (**a**) Main cutting force; (**b**) Feed force.

Figure 3 shows the cutting forces of the four types of tools. The results show that both feed force and main cutting force increase as cutting depth increased, independent of tool type. The cutting forces of micro textured tools are less than that of non-textured tools. The main cutting force and feed force of line, sinusoidal, and rhombus textured tool can be reduced by 30.27%–30.97% and 10.21%–20.61%, respectively. 

Among the four tools, the sinusoidal textured tool can produce the smallest cutting force. The explanation for this phenomenon is that the direction of the sinusoidal micro texture is nearly perpendicular to the direction of chip flow. On the one hand, it can reduce the contact area between the chip and rake face. On the other hand, it plays a beneficial role in chip breaking [28,29,30]. As a result, the sinusoidal textured tool is more suitable for the cutting of titanium alloy due to the lower cutting force. 

### 3.2. Effect of Tool Type on Chip Morphology 

**Figure 4:**
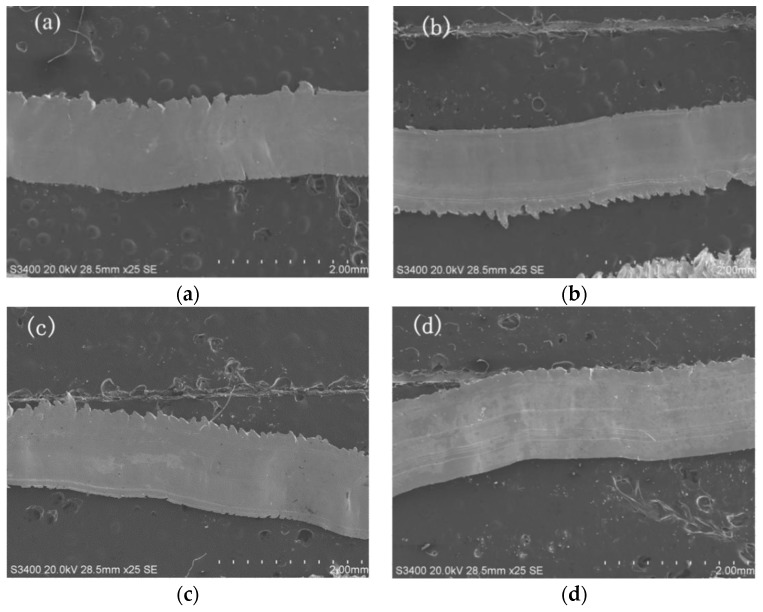
The back surface of the chips produced by the four tools. (**a**) Produced by a non-textured tool; (**b**) Produced by a line textured tool; (**c**) Produced by a sinusoidal textured tool; (**d**) Produced by a rhombic textured tool.

During the cutting process, the cutting layer of the workpiece is extruded under the action of the cutting edge and the rake face of the tool, causing shear slip deformation along the cutting surface, which is transformed into chips and forms the processed surface. The SEM image of the back surface of the chip obtained at 90.4 m/min is shown in Figure 4. The cutting layer was squeezed by the tool, which generated shear slip and eventually formed chips. The back surface of the chip was always in contact with tool and subject to a large constraint, which presented a smooth surface feature. The greater the constraint, the smoother the back surface of the chip.

**Figure 5:**
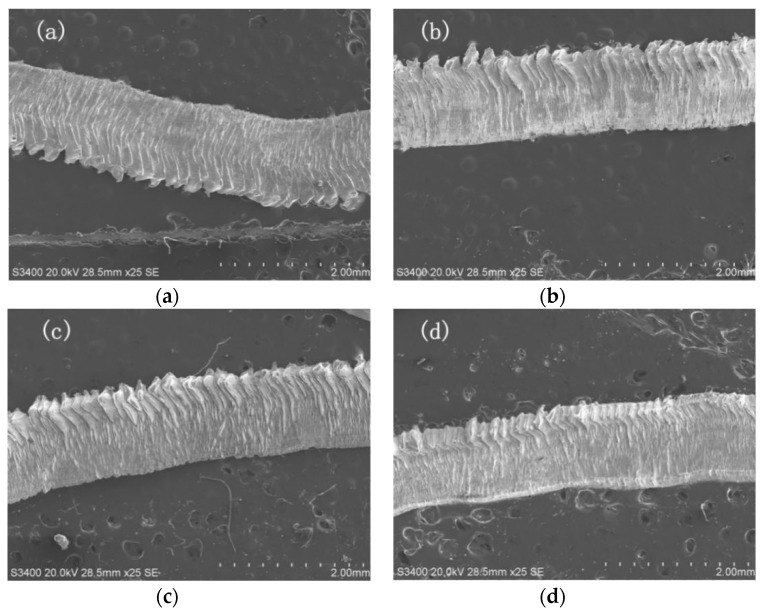
The free surface of the chips produced by the four tools. (**a**) Produced by a non-textured tool; (**b**) Produced by a line textured tool; (**c**) Produced by a sinusoidal textured tool; (**d**) Produced by a rhombic textured tool.

The SEM image of free surface at 90.4 m/min is shown in Figure 5. It can be noticed from Figure 5 that the free surface presents a pleated structure. The free surface of the chip is in a free state during the cutting process, and the restraining force is small, therefore, it would produce large deformation. As is shown in Figure 5, the free surface showed a consistent shear slip pattern while the structures were mainly sawtooth-shaped. The morphology of the chips generated by the four tools was similar. All of them were continuous chips and the two sides of the chips were serrated.

Compared with three textured tools, the sawtooth width of the non-textured tool was larger; there are cracks and more adhesive materials on the free surface. Morphology of the chips produced by the textured tool was more regular, the sawtooth structure on both sides was more uniform, and there was less adhesion on the surface. Therefore, the deformation of the chip produced by the textured tool is weaker than that produced by the non-textured tool.

The micro textures on the tool surface would cause a chip breaking effect, by which the shape of chips could effectively be controlled. Expected chips are produced during the cutting process to avoid the chip winding on the tool, so that the normal cutting processing can be guaranteed. The experimental results are of great significance to chip control during cutting. Mastery of the influence of micro texture on the chip is helpful in controlling the chip formation, shape, and size. As a result, the cutting performance would be improved by identifying chip breaking and curling chips.

### 3.3. Effect of Tool Type on the Wear Morphology of the Rake Face

**Figure 6:**
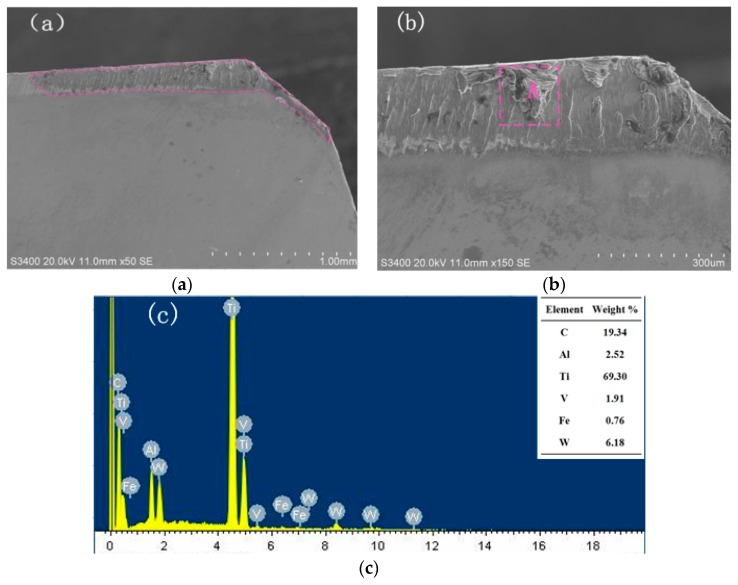
SEM and EDX of wear at the rake face of non-textured tool (*v* = 90.4 m/min, *a_p_* = 0.4 mm). (**a**) Wear morphology; (**b**) Enlarged morphology; (**c**) EDX of zone A.

The SEM image of the wear morphology of the non-textured tool and the EDX energy spectrum are shown in Figure 6. Violent wear can be observed on the wear zone of the rake face, as is shown in Figure 6b. The EDX energy spectrum shows that the main elements of the wear zones A are C and Ti. It can be inferred that the wear zone is the bonding material of the titanium alloy, where adhesive wear occurs. During the cutting process, high temperature and pressure are generated on the tool–chip contact surface due to the mutual extrusion between tool and chip. Therefore, the bottom layer of the chip would be expected to be adhered to the tool surface in this situation, which results in adhesive wear.

**Figure 7:**
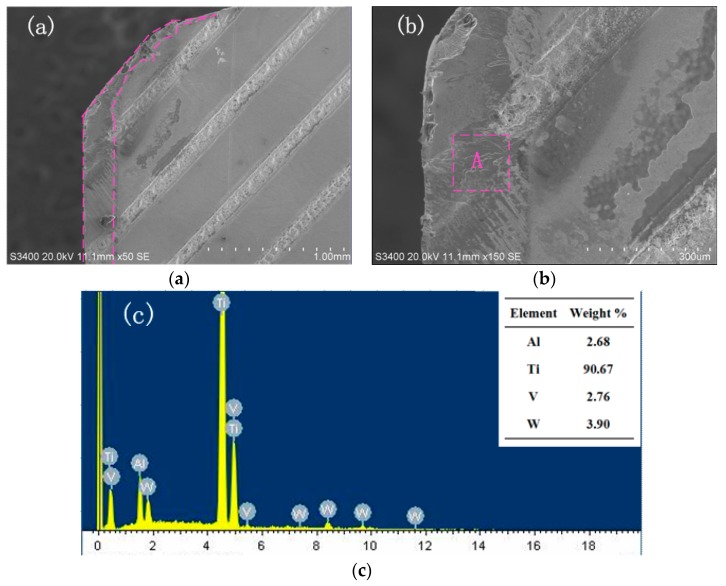
SEM and EDX of wear at the rake face of line textured tool (*v* = 90.4 m/min, *a_p_* = 0.4 mm). (**a**) Wear morphology; (**b**) Enlarged morphology; (**c**) EDX of zone A.

Figure 7 shows the wear morphology and EDX energy spectrum of the rake face of the line textured tool. Figure 7a shows that adhesion occurs on the tool rake face near the tip and the groove texture on the main cutting edge. During the cutting process, severe friction is generated on the rake face, causing high pressure and high temperature. The higher the temperature and pressure on the cutting edge, the more severe the tool wear. As a result, the texture near the cutting edge is destroyed. 

The EDX energy spectrum analysis of the groove texture zone A is shown in the Figure 7c. It demonstrates that the main element in this zone is Ti, with a content of 90.67%. It can be seen that adhesive wear occurs in zone A. Compared with non-textured tools, the wear occurring on the line textured tool is much weaker. This is because lower temperatures and lower pressures are generated on the rake face, because the presence of micro textures can store lubricating fluid which has a good cooling effect and can reduce the friction of the cutting contact surface. With the decrease of the temperature of the tool–chip interface, the adhesion of the titanium alloy becomes weaker, and the tool wear decreases [31].

**Figure 8:**
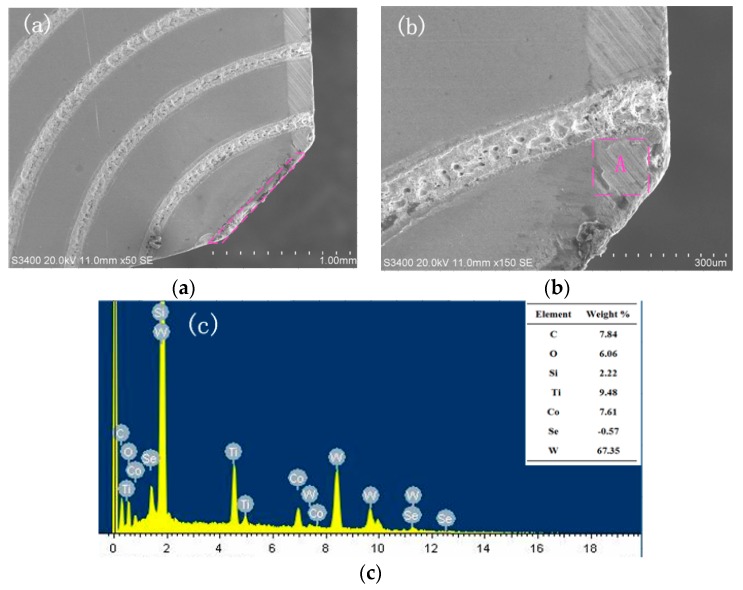
SEM and EDX of wear at the rake face of sinusoidal textured tool (*v* = 90.4 m/min, *a_p_* = 0.4 mm). (**a**) Wear morphology; (**b**) Enlarged morphology; (**c**) EDX of zone A.

Figure 8 shows the wear morphology and element energy spectrum of the rake face of sinusoidal textured tools. It can be observed from Figure 8a that the wear area of the tool rake face is small. Figure 8b shows that there is no bulk bond and the wear width is narrow. It can be noticed from Figure 8c that there is lots of element W in the wear zone, which is the base material of YG8 cemented carbide. Besides, there is some elemental O in the wear zone, which indicates that the titanium element has been oxidized with the oxygen element in the air, and oxidative wear occurred. Cutting heat is generated owing to the elastic deformation and plastic deformation of the titanium alloy, which can be as high as 800–900 °C. The average temperature of the contact area between the chip and rake face increases due to cutting heat. At a certain temperature, a chemical reaction happens between the tool material and oxygen element in the air, and a layer of a lower hardness compound is formed on the tool surface. 

**Figure 9:**
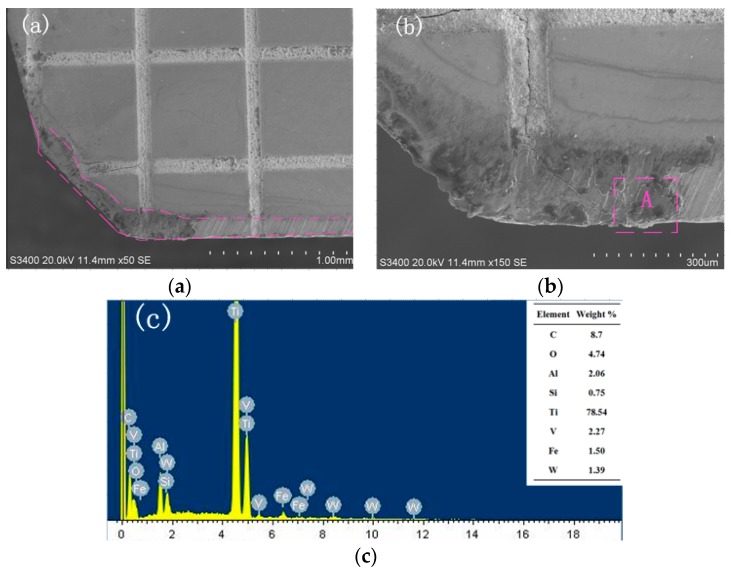
SEM and EDX of wear at the rake face of rhombic textured tool (*v* = 90.4 m/min, *a_p_* = 0.4 mm). (**a**) Wear morphology; (**b**) Enlarged morphology; (**c**) EDX of zone A.

Figure 9 shows the wear morphology and EDX energy spectrum of the rhombic textured tool. From Figure 9a, it can be observed that there is severe wear while no large clump is found in the wear zone. The EDX of zone A indicates that the wear area is mainly composed of element Ti and C, and in addition a small amount of elemental O can be found. Therefore, there are both adhesive wear and oxidative wear. 

There are several reasons for the wear phenomenon. On the one hand, temperature is an important indicator of wear and adhesion [32], and the thermal conductivity of titanium alloy is low, which causes high cutting temperature and large deformation during the cutting, resulting in tool wear. On the other hand, titanium easily reacts with other elements at high temperatures due to its strong chemical activity. Therefore, it is easy to produce a built-up edge in case of high temperature and pressure, causing a decrease in tool life. Furthermore, the melting point of elements in cemented carbide is low. The high temperature in the cutting process and the transfer of elements cause the loss of elements, resulting in tool wear.

The presence of a textured tool can lead to the decrease of tool–chip contact length, the temperature of cutting process, the work hardening of titanium alloy, and the adhesion of titanium alloy. Therefore, the wear on the tool rake face decreases in the case of micro textured tools. This study is of great significance for reducing the sticking phenomenon during cutting of titanium alloys, improving tool life, and reducing tool wear. 

### 3.4. Effect of Tool Type on the Machined Surface Roughness

**Figure 10:**
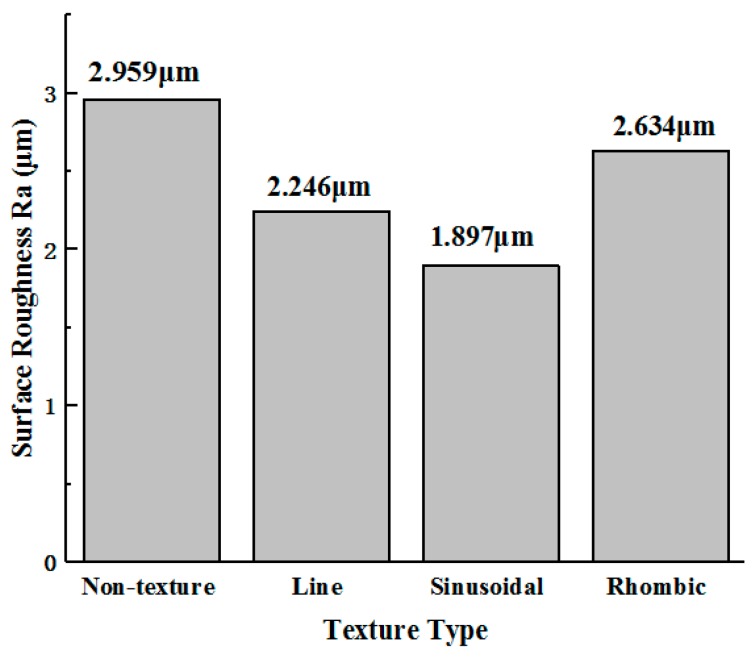
Machined surface roughness of titanium alloy (*v* = 90.4 m/min, *a_p_* = 0.4 mm).

The surface roughness Ra of Ti-6Al-4V is shown in Figure 10. The surface roughness Ra of titanium alloy machined by non-textured tools, line textured tools, sinusoidal textured tools, and rhombic textured tools is 2.959 μm, 2.246 μm, 1.897 μm, and 2.634 μm, respectively. The surface roughness Ra of the surface obtained from three types of textured tools is lower than that of the non-textured tool. Among them, the surface roughness Ra of surfaces machined by line, sinusoidal, and rhombic textured tools is reduced by 24.10%, 35.89%, and 10.98%. Compared with the other two textured tools, the surface quality of titanium alloy machined by the sinusoidal textured tool was better. Therefore, the sinusoidal textured tool is the most suitable for the machining of Ti-6Al-4V. 

## 4. Conclusions

Under the lubrication condition of cutting fluid, the experiments of cutting titanium alloy with different cutting speeds and depths were carried out using YG8 tools with line, rhombic, and sinusoidal groove textures on the rake face. The main conclusions were as follows:

(1) Under the same lubrication condition, three types of texture on the tool rake face were all effective in reducing cutting force and tool wear. Among the three types of textured tools, the sinusoidal textured tool caused the best cutting performance, followed by the line textured tool and rhombic textured tools.
(2) The decrease of main cutting force can reach up to 30.97% by using textured tools. The morphology of chips produced by textured tools is better than that produced by non-textured tools. The existence of the textures on the tool surface is beneficial for chip breaking.
(3) On the rake face of the non-textured tool appears violent adhesive wear, while adhesive wear of the textured tool is weaker. The sinusoidal textured tool shows the best anti-adhesion effect among the four kinds of tools.
(4) The roughness of the machined surface produced by textured tools is significantly lower than that of the non-textured tool. Titanium alloy machined by the sinusoidal textured tool has the lowest surface roughness, which reduced by 35.8% compared with that of the non-texture tool.

## Figures and Tables

**Table 1:** Physical characteristics of the YG8 material.

Material	Hardness (HRA)	Density (g/cm^3^)	Elastic Modulus (GPa)	Bending Strength (MPa)	Thermal Conductivity (W/mk)
YG8	89	14.6	630	1840	79.6

**Table 2:** Geometric parameters of the tool.

Rake Angle *γ_o_*	Inclination Angle *λ_s_*	Clearance Angle *α_o_*	Side Cutting Edge Angle *k_r_*
6°	−6°	11°	75°

**Table 3:** Parameters of laser processing.

Wave Length (nm)	Frequency (KHz)	Power (W)	Pulse Width (ns)	Scanning Speed (mm/s)	Repetitions
1064	20	40	20	100	200

**Table 4:** The experimental cutting parameters.

Number	Cutting Speed *v* (m/min)	Cutting Depth *a_p_* (mm)	Feed *f* (mm/r)	Cutting Time (s)
1	22.7	0.3	0.2	160
2	47.7	0.3	0.2	160
3	71.9	0.3	0.2	160
4	90.4	0.3	0.2	160
5	47.7	0.1	0.2	160
6	47.7	0.2	0.2	160
7	47.7	0.3	0.2	160
8	47.7	0.4	0.2	160
